# Different heart failure phenotypes of valvular heart disease: the role of mitochondrial dysfunction

**DOI:** 10.3389/fcvm.2023.1135938

**Published:** 2023-05-19

**Authors:** Shenghui Zhang, Cheng Liu, Yingyuan Zhang, Zongjian Wu, Kaiwei Feng, Yanxian Lai, Jingxian Pei, Tianwang Guan

**Affiliations:** ^1^ Department of Cardiology, The Second Affiliated Hospital, School of Medicine, South China University of Technology, Guangzhou, China; ^2^ Department of Cardiology, Guangzhou First People’s Hospital, South China University of Technology, Guangzhou, China; ^3^ Department of Cardiology, Guangzhou First People’s Hospital, Guangzhou Medical University, Guangzhou, China; ^4^ Department of Cardiothoracic Surgery, The First Affiliated Hospital of Guangzhou Medical University, Guangzhou, China; ^5^ City School, Guangzhou Academy of Fine Arts, Guangzhou, China; ^6^ Department of Cardiology, The Second Affiliated Hospital of Guangzhou Medical University, Guangzhou, China

**Keywords:** valvular heart disease (VHD), valvular damages, heart failure, phenotypes, mitochondrial dysfunction

## Abstract

Valvular heart disease (VHD)-related heart failure (HF) is a special subtype of HF with an increasingly concerned heterogeneity in pathophysiology, clinical phenotypes, and outcomes. The mechanism of VHD-related HF involves not only mechanical damage to the valve itself but also valve lesions caused by myocardial ischemia. The interactions between them will lead to the occurrence and development of VHD-related HF subtypes. Due to the spatial (combination of different valvular lesions) and temporal effects (sequence of valvular lesions) of valvular damages, it can make the patient's condition more complicated and also make the physicians deal with a dilemma when deciding on a treatment plan. This indicates that there is still lack of deep understanding on the pathogenic mechanism of VHD-related HF subtypes. On the other hand, mitochondrial dysfunction (MitD) is not only associated with the development of numerous cardiac diseases such as atherosclerosis, hypertension, diabetes, and HF but also occurs in VHD. However, the role of MitD in VHD-related HF is still not fully recognized. In this comprehensive review, we aim to discuss the current findings and challenges of different valvular damages derived from HF subtypes as well as the role of MitD in VHD-related HF subtypes.

## 1. Introduction

Valvular heart disease (VHD) is characterized by damage and dysfunction in one or more of the four valves of the heart, due to inflammation, myxoid degeneration, and other reasons. VHD becomes one of the major global health problems, showing different disease burdens in developing and developed countries (1). In developing countries such as Africa, Pacific Island countries, and Asia, rheumatic VHD is the most prevalent subtype of VHD, its morbidity and mortality remain high, and it is still overlooked. In most developed countries, degenerative VHD is mainly the prevalent subtype of VHD, and its related morbidity and mortality have been increasing in elderly patients over the past two decades. Heart failure (HF) is a group of complex clinical syndromes caused by cardiac functional and/or structural disorders for impairing ventricular filling or ejection fraction (EF) and finally resulting in insufficient cardiac output inability to meet the metabolic needs of body tissues. HF is divided into three subtypes based on the cutoff value of left ventricular EF (LVEF), such as heart failure with reduced ejection fraction (HFrEF), heart failure with preserved ejection fraction (HFpEF), and heart failure with mildly reduced ejection fraction (HFmrEF). As the most common complication of VHD, HF is its leading cause affecting the survival and quality of life in VHD patients (2, 3). Unlike HF which is caused by coronary artery disease, hypertension, diabetes mellitus, and/or other reasons, VHD-related HF is recognized as a special subtype of HF, and its clinical manifestations and outcomes are still so far not well understood, especially the relationships of various valvular damages with the three HF subtypes.

Mitochondria (Mit), as double-membrane-bound organelles, are found in almost all eukaryotic cells and primarily located within subsarcolemmal, perinuclear, and intrafibrillar regions of the cardiomyocyte (4). The major function of Mit is to generate large quantities of energy. Aside from energy production, Mit also regulate cell signaling transduction, generate heat, and mediate cell growth and death (5). Mitochondrial dysfunction (MitD), including altered metabolic substrate utilization, impaired mitochondrial oxidative phosphorylation, increased reactive oxygen species formation, and aberrant mitochondrial dynamics (6), is not only associated with the development of numerous cardiac diseases such as atherosclerosis, hypertension, diabetes, and HF (7) but also occurs in VHD (8). However, the role of MitD in VHD-related HF is still not fully understood.

This review provides a discussion of the current findings and challenges related to different valvular damages derived from HFrEF, HFmrEF, and HFpEF, as well as the role of mitochondrial abnormalities in VHD-related HF subtypes.

## 2. Left heart valve damages associated with HFrEF

HFrEF is defined as LVEF of less than 40% (3), while HFmrEF is characterized by an LVEF between 40% and 50% along with mild systolic dysfunction (SD) and diastolic dysfunction (DD). Given the similarities on etiology and outcomes between HFrEF and HFmrEF, this review will use the term HFrEF to refer to the combined subtypes unless specified otherwise. While the incidence of HFrEF has been decreasing over the years, it remains the predominant subtype of HF, accounting for 63%–84% of all HF cases. Recent studies revealed that VHD is responsible for 5.0%–13.6% of HFrEF cases, which can be attributed to valvular damages and its related myocardial ischemia (9–11).

### 2.1. Mitral valve damages and HFrEF

Mitral valve damages increases the risk of HFrEF. A recent study on evaluating the associations of cardiac valve damages in a Lebanese population with myocardial function reported that mitral valve diseases were associated with a 6.2-fold increased risk of left ventricular (LV) SD but exhibited heterogeneity with different types of valvular damages (12). Mitral regurgitation (MR) alters contractility by inducing LV remodeling, while mitral stenosis (MS) directly reduces LV strain and contractility. An epidemiological study on VHD in Europeans found that pure MR progressed to HFrEF in approximately 19.7% of patients (13). The echocardiographic features of MR patients with HFrEF is more likely to present LV eccentric remodeling with MR deterioration and ultimately result in SD and significantly decreased EF (14). The core mechanism of developing HFrEF in MR patients is myocardial fibrosis. A study on exploring abnormal gene expression in MR patients demonstrated that there were subclinical myocardial structural and functional abnormalities in the procession from primary MR to HFrEF with new-onset clinical symptoms, showing the characteristics such as LV end-systolic volume gradually enlarged and LVEF gradually or drastically decreased in this process. At the same time, myocardial profibrotic genes were overexpressed, then promoted extracellular interstitial protein deposition, and finally led to exacerbation of myocardial fibrosis (15). In a rat model of MR, similar pathological changes were observed (16), which were manifested as increased LV myocardial mass and myocardial cell length, decreased myocardial thickness, and sarcomere disorder/interruption, resulting in decreased global myocardial contractility.

Despite lack of clinical epidemiological studies on MS and HFrEF, clinical ultrasound studies suggested that MS potentially increased the risk of HFrEF. Snyder et al*.* (17) found that approximately 30% of patients with pure MS had an increased LV volume as well as a decreased LVEF because of LV SD. A spot-tracking study of subclinical MS revealed that MS patients were prone to LV SD compared with normal individuals, presenting as decreased LV strain and LV strain rate, and exacerbated with MS deterioration (18). Of note, the LV strain was significantly decreased in patients with mild MS, suggesting that there was subclinical LV SD even in the early stage of MS. Mangoni et al. (19) also found that the LV end-diastolic diameter enlarged in MS patients with reduced EF compared with those with normal EF. These findings indicated that MS can cause LV SD, and the main mechanisms involved are as follows (17, 19): (I) LV diastolic filling insufficiency; (II) the etiology of MS, such as rheumatic VHD, which directly caused the reduction of LV contractility and strain; and (III) right ventricular (RV) enlargement-induced regional dysfunction of LV. The effect of MS on myocardial function was related to myocardial remodeling caused by local or systemic inflammation and also to ischemic damage (e.g., myocardial infarction) (20).

### 2.2. Aortic valve damages and HFrEF

Aortic stenosis (AS) is the most common cause of HFrEF, accounting for approximately 6.0%–35.0% of patients with HFrEF. Among AS patients with HFrEF, the clinical phenotype was presented in males, a history of myocardial infarction and diabetes mellitus, which was similar to that of HFrEF patients without valvular damages (13, 21). During the five development stages of AS progression, the left heart SD (LVEF < 50%) may present even in early stage of AS (22). In mild to moderate AS patients, once LVEF was of <60%, the stenosis degree of aortic valve dramatically deteriorated within 3 years and correlated with a rapid progression of HF, which could be used as a predictor for clinical intervention in AS patients (23). In a pig model of severe AS, Ishikawa et al. (24) found that there was no SD in the early stage of course development, but there was lower LV–arterial compliance as well as higher LV stiffness and had a tendency to rapidly progress to HF. Jean et al. (25) reported that HFrEF patients with moderate AS had a three-fold increase risk of death compared with those without AS, and this risk cannot be improved even with aortic valve replacement. The findings indicated that the clinical characteristics of AS patients with HFrEF were manifested with early onset, rapid progression, and poor prognosis.

In clinical practice, low-gradient AS (LG-AS) was divided into three types, namely, normal-flow LG-AS, paradoxical low-flow LG-AS, and classic low-flow LG-AS (26). Classic low-flow LG-AS represents the HFrEF form of AS, indicating the characteristics defined to be LVEF of <50%, aortic valve area of ≤1.0 cm^2^, and mean gradient of <40 mmHg (27). Low flow was associated with SD due to severe AS and/or other diseases (e.g., ischemic cardiomyopathy). Low pressure gradient was associated with a decreased stroke volume due to reduced longitudinal strain, resulting in lower transvalvular gradient (28). Notably, the appearance of classic low-flow LG-AS indicated a valve, vascular, and myocardial dysfunction due to severe fibrosis (29), which was associated with the worst prognosis compared with normal-flow and paradoxical low-flow LG-AS. Furthermore, the QRS complex on an electrocardiogram represents ventricular depolarization, and its duration usually ranges from 0.08 to 0.10 s. A widened QRS duration (exceeding the normal range) can indicate ventricular conduction delays or ventricular dyssynchrony. The increased QRS duration occurred in patients with classic low-flow LG-AS (30). This suggested that those patients suffered from cardiac conduction disturbances and poor synchronization of ventricular contraction and filling, which was at least partially associated with left and right heart SD (30). Consistent with this study (30), Ito et al*.* (28) and Henkel et al*.* (31) also found that the risk of left bundle branch block significantly increased in severe AS patients with HFrEF compared with those with normal LVEF, which was a key marker of the occurrence of LV SD in severe AS patients.

The pathophysiological mechanisms of AS-induced left heart dysfunction involves increased LV afterload, intermittent ischemia, neurohumoral activation, abnormal contractility of microtubules within myocyte cytoskeleton, and abnormalities in calcium handling (32, 33). Of these, the crucial mechanism was the increase of LV afterload, related to LV concentric remodeling. This is the most common LV adaptive remodeling in AS, manifesting to be ventricular hypertrophy to overcome increased afterload and also exacerbate myocardial energy consumption. Pibarot et al. (34) reported that AS patients were predisposed to HFrEF after LV concentric remodeling. In the early stage, an increase in LV afterload is caused by LV concentric remodeling, which worsen coronary hypoperfusion, induced myocardial ischemia, and promoted myocardial fibrosis, eventually resulting in LV SD and clinical symptoms of HF (21). With the progress of AS, “eccentric remodeling” occurred in moderate AS patients with HFrEF, which was associated with increased all-cause mortality (35). It is worth noting that “eccentric remodeling” was the end-stage manifestation of LV remodeling in AS patients, caused by a combination of LV SD and increased LV afterload (36, 37). On the other hand, the presence of LV concentric/eccentric remodeling implicated a myocardial energy metabolism imbalance. A meta-analysis explored myocardial efficiency in patients with different etiologies and stages of HF and demonstrated that myocardial contractility progressively decreased with the reduction of myocardial efficiency in AS patients symptomatic HFrEF (38). The reduction of energy utilization efficiency was caused by decreased creatine kinase activity and phosphocreatine/adenosine triphosphate ratio in AS patients (38, 39). These findings indicated that AS patients had not only hemodynamic abnormalities resulting from mechanical damage to the valves but also had damage of myocardial function. This may partially explain why pure aortic valve replacement did not reduce the long-term prognosis of AS patients. Hence, combination therapy by comprehensive drug therapy and valve replacement may be the preferred strategy to improve the prognosis of AS patients with HFrEF.

In contrast to AS, the progression process of aortic regurgitation (AR) is relatively slow. Patients with mild to moderate AR may have no obvious clinical symptoms. Once progressing to severe AR, the myocardial function is impaired (e.g., decreased myocardial compliance), and clinical symptoms of HF rapidly occur (40). The study of the Euro Heart Survey on VHD showed that about 24.5% of AR patients developed HFrEF (13). The proportion was up to 38.6% in China, mainly related to degenerative valve damages (41). AR patients with HFrEF had a poor prognosis, and the mortality was up to 74% within 10 years (42). It is worth noting that, unlike the non-VHD-related HFrEF (LVEF of <40%), LVEF of <55% was considered as LV SD in AR patients according to the 2020 AHA/ACC guidelines on VHD (43). A recent study by Zhao et al*.* (41) found that AR patients with LVEF of <55% had an increased risk of a 2-year mortality or HF rehospitalization, and the effectiveness of drug treatment was significantly weakened in those patients, suggesting that LVEF of <55% may be an optimal predictive marker for intervention and prognosis in AR patients.

The progression of AR to HFrEF is actually a process of compensation to decompensation of LV adaptation to regurgitated blood volume. During this process, AR patients may not exhibit any evident symptoms and/or signs of HF for an extended period. In the compensatory phase, elevated preload leads to LV concentric/eccentric remodeling, particularly the eccentric remodeling, which gradually results in extensive interstitial fibrosis and collagen composition changes. In the decompensated phase (manifested by increased wall pressure), LV compliance was reduced in AR patients, resulting in HFrEF. On the other hand, increased LV afterload reduces coronary perfusion, causing subendocardial ischemia that leads to irreversible replacement of necrotic cardiomyocytes with the fibrous tissue and resulting in LV SD and poor prognosis in AR patients (33, 44, 45). Bussoni et al. (46) constructed a rat model of AR and observed the following LV structural changes during the process of AR to HFrEF: spherical LV remodeling appeared within 1 week, eccentric remodeling significantly exacerbated within 4 weeks, and LV SD occurred after 12 weeks. Similar eccentric remodeling phenomena were observed in chronic severe AR patients. This remodeling was well-adapted to increased wall pressure but eventually resulted in LV SD.

## 3. Left heart valve damages associated with HFpEF

HFpEF is a cluster of clinical syndromes with symptomatic HF and LVEF of ≥50%, characterized by DD of myocardial infarction (47). Approximately 34%–52.5% of patients with HF had HFpEF, and the ratio gradually increased over the years (48). Given the heterogeneity of HFpEF, there is still a deficiency of standardized and validated treatment strategies, and more hope for phenotype-guided treatment strategies on HFpEF has been anticipated (49–51). Among the HFpEF subtypes, aside from “garden variety (e.g., hypertension, diabetes mellitus, obesity, etc.),” coronary artery disease, atrial fibrillation (AF), and right heart failure (RHF)-related HFpEF (52), VHD-related HFpEF is a novel HFpEF subphenotype (53, 54). Indeed, a multicenter study in a Vietnamese population found that VHD was one of the common causes of HFpEF. These patients were younger and had fewer comorbidities, but they had earlier cardiovascular events and worse prognosis (55). Accumulating evidence showed that the incidence of VHD-related HFpEF ranged from 11.0% to 86.8% (9, 51, 55–57). VHD-related HFpEF was mainly caused by left heart valve damage, especially AS (58). A recent study revealed that the three most prominent pathophysiological features in patients with preclinical HFpEF were as follows: (I) left atrial failure (LAF), (II) pulmonary hypertension (PH) and right ventricular dysfunction (RVD), and (III) renal failure. The common mechanism was systemic chronic inflammation in these pathophysiological processes (59). Cardiovascular diseases that caused the three pathophysiological processes mentioned above and trigger systemic chronic inflammation may eventually lead to HFpEF.

### 3.1. Mitral valve damages and HFpEF

The most prominent feature of HFpEF is DD, which can be caused by both MS and MR, suggesting that patients with MS and/or MR may suffer from HFpEF. MS significantly increases the risk of LV DD and mostly develops in elderly patients and/or in patients with hypertension, which is consistent with the etiological features of HFpEF (12). LAF is the critical pathophysiological process in the early stage of HFpEF in MS patients. In MS patients, increased left atrial (LA) pressure, which induced LA eccentric remodeling, simultaneously resulted in LA pumping dysfunction and eventually led to LAF when decompensation occurred (60). On the other hand, MS patients are predisposed to AF (61), relating to the electrical remodeling on the basis of structural remodeling. AF, as one of the prominent clinical features of LAF, is an initial sign of progression to HFpEF in MS patients (59). LAF and AF interact to form a vicious circle. LV DD also occurs with the thickening or restricting activity of the mitral valve ring in MS patients, which is another potential pathogenesis of MS-induced HFpEF (62). In addition, Hoshida et al. (63) demonstrated that indicators for assessing LA pressure overload, such as the ratio of diastolic elasticity to arterial elasticity, could be used for evaluating the prognosis in patients with HFpEF rather than LA volume overload.

MR also increases the risk of HFpEF. In the Lebanese population, Nader et al. (12) found that MR increased the risk of LV DD, even in MR patients who did not have HF symptoms, but still had a probability of progression to HFpEF. MR patients with LV DD often suffer from hypertension, coronary artery disease, and diabetes mellitus, consistent with the etiological characteristics of HFpEF (62). Compared with HFpEF patients without MR, HFpEF patients with MR had worse biventricular function and hemodynamics (64). During the period from the compensated stage of MR to MR with HFpEF, the functional and/or structural abnormalities of LA are the major clinical features. Increased regurgitated blood volume due to MR resulted in eccentric remodeling of LA and LV, successively (62, 65). Yang et al. (65) showed that increased regurgitated blood volume resulted in LA eccentric remodeling as well as the reduction of LA compliance, which is involved in the pathophysiological process of chronic inflammation and LA hypertrophy. Hence, the occurrence of HFpEF can be effectively predicted by global peak positive strain of the left atrium and strain rate in the LA filling phase. Functional MR in patients with HFpEF reflects LA myopathy and was correlated with worse hemodynamic status and poorer cardiac capacity, independent of rhythm (e.g., AF) (64). The recent study reported that the prevalence of AF increased with MR severity and the interaction between AF and MR deteriorated the patient outcome (66). This harmful effect was more pronounced in HFpEF compared with HFrEF. The potential mechanism was correlated with increasing regurgitated blood volume-induced LA eccentric remodeling and then LAF, which mediated the occurrence and development of MR-related HFpEF. In addition to LA eccentric remodeling, MR also resulted in LV eccentric remodeling. With the progression of MR, the myocardium of MR patients becomes stiff under the etiology of HFpEF as mentioned above, and mild MR can cause an abnormal increase of LV filling pressure and finally lead to LV eccentric remodeling, which further increases LV filling pressure to form a vicious circle. LV eccentric remodeling occurs in MR patients, but LVEF is still normal in a certain period (58). In addition, exercise-induced MR is defined as a development of at least moderate MR during exercise. In HFpEF patients, exercise-induced MR is commonly present and shows a distinct phenotype characterized by better chronotropic reserve and peripheral oxygen extraction (67).

### 3.2. Aortic valve damages and HFpEF

AS increases the risk of HFpEF. In clinical practice, paradoxical low-flow LG-AS represents the HFpEF form of AS. Epidemiological studies showed that this AS subtype accounted for approximately 5%–15% of all AS categories and is most commonly found in elderly patients, in women, and in patients with hypertension, coronary artery disease, AF, and/or diabetes mellitus (26, 27, 58). The significant echocardiographic feature of patients with this AS subtype is low transaortic flow with preserved LVEF, which is characterized by severe stenosis (aortic valve area of ≤1 cm^2^), low gradient (mean gradient of <40 mmHg), low flow (peak aortic velocity of <4 m/s), and LVEF (>50%) (27, 68). Paradoxical low-flow LG-AS was associated with an intermediate prognosis compared with normal-flow LG-AS with a good prognosis and classic low-flow LG-AS with the worst prognosis. Valvular damage itself and its related myocardial fibrosis are the core pathophysiological link in the progression of AS to HFpEF, involving systemic and/or local chronic persistent low-grade inflammation (58). The thickness of the epicardial adipose tissue increases in AS patients, promoting the release of systemic inflammatory factors and the occurrence of chronic inflammation (69). AS-induced pressure overload also triggers the release of inflammatory mediators, resulting in the occurrence and development of myocardial fibrosis (70). AS-related myocardial concentric remodeling may cause microvascular dysfunction and ultimately lead to DD (71, 72). Indeed, a recent study on unsupervised statistical learning for identifying the phenogroups of LV DD found that patients with mild and moderate AS had an increased LV DD risk due to AS-induced moderate to severe myocardial remodeling, predisposing to HFpEF (73).

The relationship between AR and HFpEF remains not fully understood. LV eccentric remodeling (e.g., increased diameter/volume ratio and mass) can appear in the early stage of AR. With the development of LV eccentric remodeling, LV compliance decreased accordingly. Once the LV eccentric remodeling cannot compensate to the harmful effect of AR-induced regurgitated blood volume, the end-diastolic pressure evidently increased and then HF-related symptoms followed, which suggested that AR patients had the potential to develop HFpEF (62). Zhang et al*.* (74) revealed that DD presented in patients with bicuspid aortic valve, correlated with decreased hoop compliance due to AR. Notably, AR-induced regurgitation was counterclockwise, which may counteract forward (clockwise) pumping during ventricular diastole, resulting in impaired LV filling. This counteracting effect was more evident in patients with regurgitation biased to the posterior leaflets of the mitral valve, which restricted the diastolic opening of the mitral anterior leaflets. Under some circumstances, it will lead to severe MR, insufficient LV pumping, and myocardial diastolic dysfunction, showing as decreased hoop and longitudinal diastolic strain rates (75). AR increased the risk of poor prognosis in HFpEF patients. Bolat and Biteker (76) previously found that the 1-year all-cause mortality and HF rehospitalization significantly increased in HFpEF patients with AR. Abdurashidova et al*.* (77) recently showed that even mild AR was associated with a two-fold increase risk on short-term all-cause mortality in HFpEF patients. Inconsistent with the above findings, Nader et al*.* (12) reported that AR was a protective factor for the development of DD among VHD patients in the Lebanese population, which may be related to study selection bias.

## 4. Right heart valve damages associated with HF

The prevalence of right heart valve damages in the adult population is significantly lower than that of left heart valve damages. Right heart valve damages (e.g., stenosis and regurgitation) included tricuspid and pulmonary valve lesions. Tricuspid stenosis, pulmonary stenosis, and pulmonary regurgitation are rare valve defects, especially the first two. Thus, in this section, we will only discuss the tricuspid regurgitation (TR)-related HF.

EF, as a key measure of cardiac systolic function, is categorized into LVEF and right ventricular ejection fraction (RVEF). It, in the narrow sense, means the LVEF, which only reflects the systolic function status of the left heart. The aforementioned types of HF (e.g., HFrEF, HFmrEF, and HFpEF) are classified according to LVEF. However, it is worth noting that cardiac function is an organic whole and the functional status of the left heart is also influenced by the right heart. Melo et al*.* (78) first proposed the concept of RV-related HFpEF. Desai et al*.* (79) further showed that a RVEF value was positively correlated with that of the LVEF ones in patients with HFrEF. A similar phenomenon was also observed in HFpEF patients (80, 81). In the Atherosclerosis Risk in Communities Study, Nochioka et al*.* (82) revealed that an RVEF value was also decreased with reducing LVEF in the population without HF. A lower RVEF value was associated with a higher risk of HF and a worse prognosis. This adverse effect was independent of LV functional status (83). Compared with changes in LV, the structure and function of the RV deteriorate to a greater extent over time (84). These findings indicated that the right heart function status may also be classified according to RVEF in patients with HFrEF, HFmrEF, and HFpEF. As an independent HF effect, the functional status of the right heart in various HF subtypes remains overlooked due to the lower incidence of right heart dysfunction, the relatively unobvious clinical manifestations, and insufficient diagnostic methods compared with those for left heart dysfunction.

Aside from RVEF, tricuspid annular plane systolic excursion (TAPSE) and pulmonary artery systolic pressure (PASP) can also be used to evaluate the RV function. TAPSE is the total longitudinal displacement of the tricuspid annulus from tele-diastole to end-systole, which is a powerful indicator of RV function. A TAPSE measurement of less than 17 mm indicates RVD (85), and less than 14 mm indicates a poor prognosis in patients with chronic heart failure (86). PASP is mainly used as a diagnostic indicator of PH. PASP of >35 mmHg is used to predict the occurrence of RVD and to some extent used as a predictive marker for the occurrence of potential secondary TR (85). The prevalence and phenotype of RVD in HFrEF, HFmrEF, and HFpEF showed heterogeneity and mainly depended on the relationship between TAPSE and PASP (87, 88). (I) RVD in HFrEF: approximately 60% of HFrEF patients suffered from RVD, and more than half of these patients had PH (87). However, among the HFrEF patients, the incidence of RVD was not associated with the presence or absence of PH, but with whether to combine AF and coronary artery disease or not (88). It indicated that LV SD was one of the pivotal links for the RVD occurrence in HFrEF patients under the influence of AF and coronary artery disease. A possible explanation was that the myocardial fibers between the two ventricles interacted with each other, dysfunction of one ventricle impacted the function of the other, and ischemia increased the SD risk (87). (II) RVD in HFpEF: approximately 30%–40% of HFpEF patients had RVD (87). Unlike HFrEF, PH significantly was associated with an increased RVD risk in HFpEF patients (88). A possible explanation was that PH impaired the coupling between LV and RV during diastole. Once RVD occurred, it will result in LV DD and finally progress to HFpEF (89). The TAPSE/PASP ratio is a noninvasive index of right ventricle–pulmonary artery coupling and emerges as a strong predictor of recurrent hospitalizations in HFpEF (90). Right ventricle–pulmonary artery uncoupling (RV–PAU) was independently associated with adverse outcomes in acute decompensated patients with HFpEF (91). (III) RVD in HFmrEF: HFmrEF patients had similar treatment outcomes with the HFrEF ones, but the clinical risk factors (e.g., elderly, female, hypertension, diabetes mellitus) (92) and pathophysiological features (e.g., PASP) (88) of RVD in HFmrEF patients were closer to those of patients with HFpEF. Since LV dysfunction is present in HFrEF patients, regardless of the presence of PH, RVD is more likely to occur when the compensatory reserve of RV to load was reduced. The right ventricular systolic function still had a certain compensatory capacity in the early stage among HFpEF patients. As the PH gradually increased, it will cause the imbalance of RV–PAU, which could eventually lead to the development of RVD. As far as the HF patients were concerned, better status of RV-PA coupling at baseline suggested that the heart is at a lower level of LV-induced PH and secondary RVD. If active intervention is implemented at this stage, it will be conducive to the reversion of LV remodeling and improve prognosis. Hence, RV–PAU may be the crucial determinant of RVD phenotypic heterogeneity between the three HF subtypes.

RV–PAU mainly emphasizes the influence of the interaction between right ventricular systolic function and PH status on RVD. However, the effect of the interaction between right heart valve damage and PH status on RVD is ignored, especially TR. Tricuspid valve is the core physical component of the right heart system, and its regurgitation will lead to RVD. TR can be divided into three types: primary, secondary, and isolated TR (93, 94). The main causes of primary TR are congenital heart disease, tricuspid valve prolapse, and rheumatic VHD. Isolated TR is a subtype of TR under the condition without PH and left heart disease, which may be related to right atrial dilation because of AF. The causes of secondary TR, as the most common subtype, are mainly divided into the following two categories: pressure overload (e.g., PH) and volume overload (e.g., atrial septal defect and pulmonary regurgitation). The mechanism of TR-induced RVD was directly owing to the increased preload of RV, which will result in the inability of the RV to supply adequate circulating blood volume (95). It can also be indirectly due to dysfunction of LV and RV, mediating through the interventricular septum and pericardium (96, 97). On the one hand, severe TR led to increased RV volume overload and then shifted the interventricular septum to the left. Under the circumstances, it will help reduce the LV preload but increase the LV end-diastolic pressure, which will result in LV SD, and can cause or aggravate HFrEF (96). Due to the connected myocardial fibers between the right and left heart, myocardial fibers redistributed after volume overload. This redistribution will help maintain the normal volume loading of the ventricle but can cause SD. On the other hand, TR led to RVD by increasing the RV volume overload (98). Since the right and left hearts are in the same pericardium, TR may result in a pathophysiological process for RV similar to pericardial stenosis, which would mediate the occurrence of RV DD and then increase the HFpEF risk (97). According to the origin of TR, TR is classified as either ventricular functional TR or atrial functional TR. Ventricular functional TR is defined as the presence of RV systolic pressure of >50 mmHg or RV dilation, and the remaining patients are classified as having atrial functional TR if they had RA dilation or tricuspid annular enlargement (99). TR in HFpEF is related to RA remodeling, and the presence of atrial functional TR was associated with poor clinical outcomes. Transcatheter tricuspid valve edge-to-edge repair may be promising for improved outcomes in TR patients with HFpEF (100).

## 5. Role of MitD in various valvular damages derived from HF subtypes

**Figure 1:**
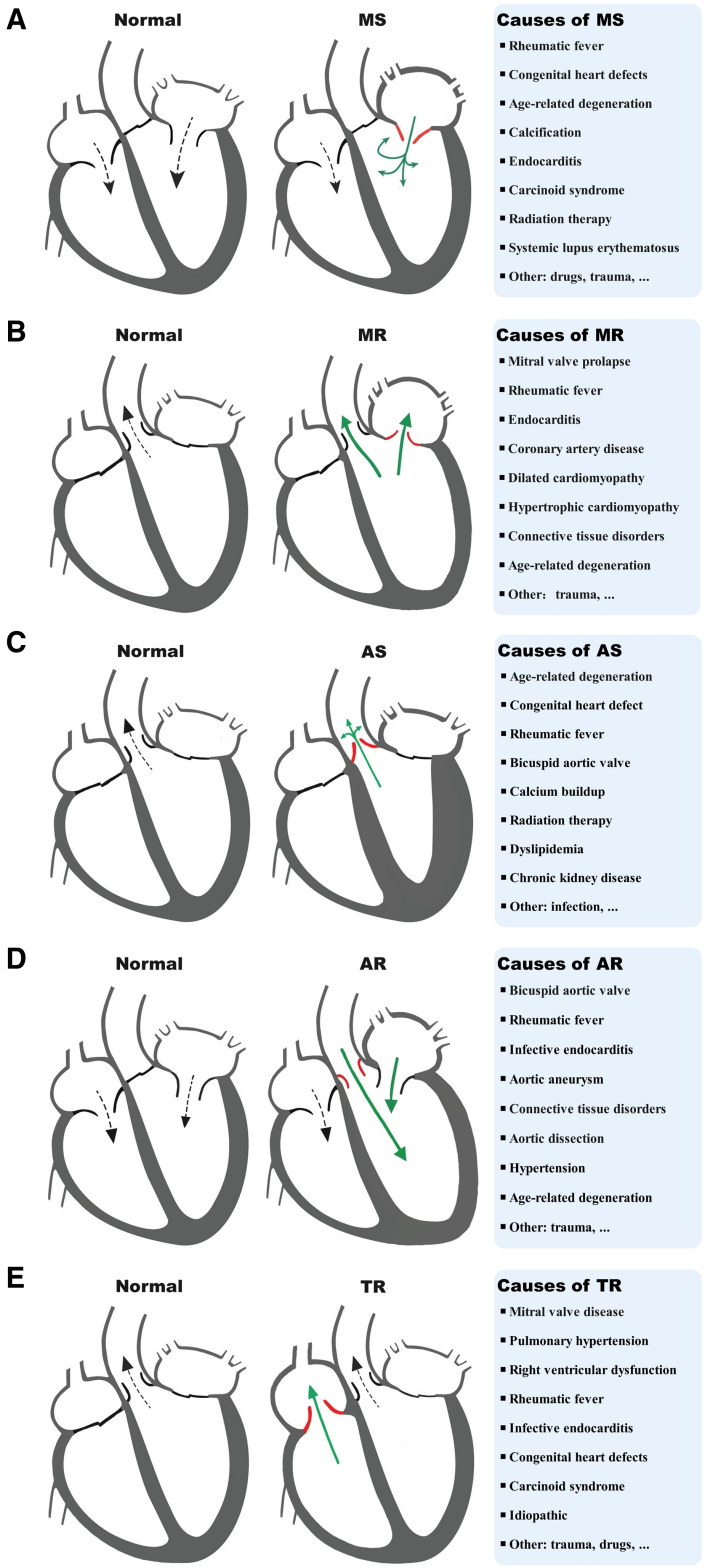
Diagram of a typical blood flow and causes of MS (**A**), MR (**B**), AS (**C**), AR (**D**), and TR (**E**). MS, mitral stenosis; MR, mitral regurgitation; AS, aortic stenosis; AR, aortic regurgitation; TR, tricuspid regurgitation.

**Figure 2:**
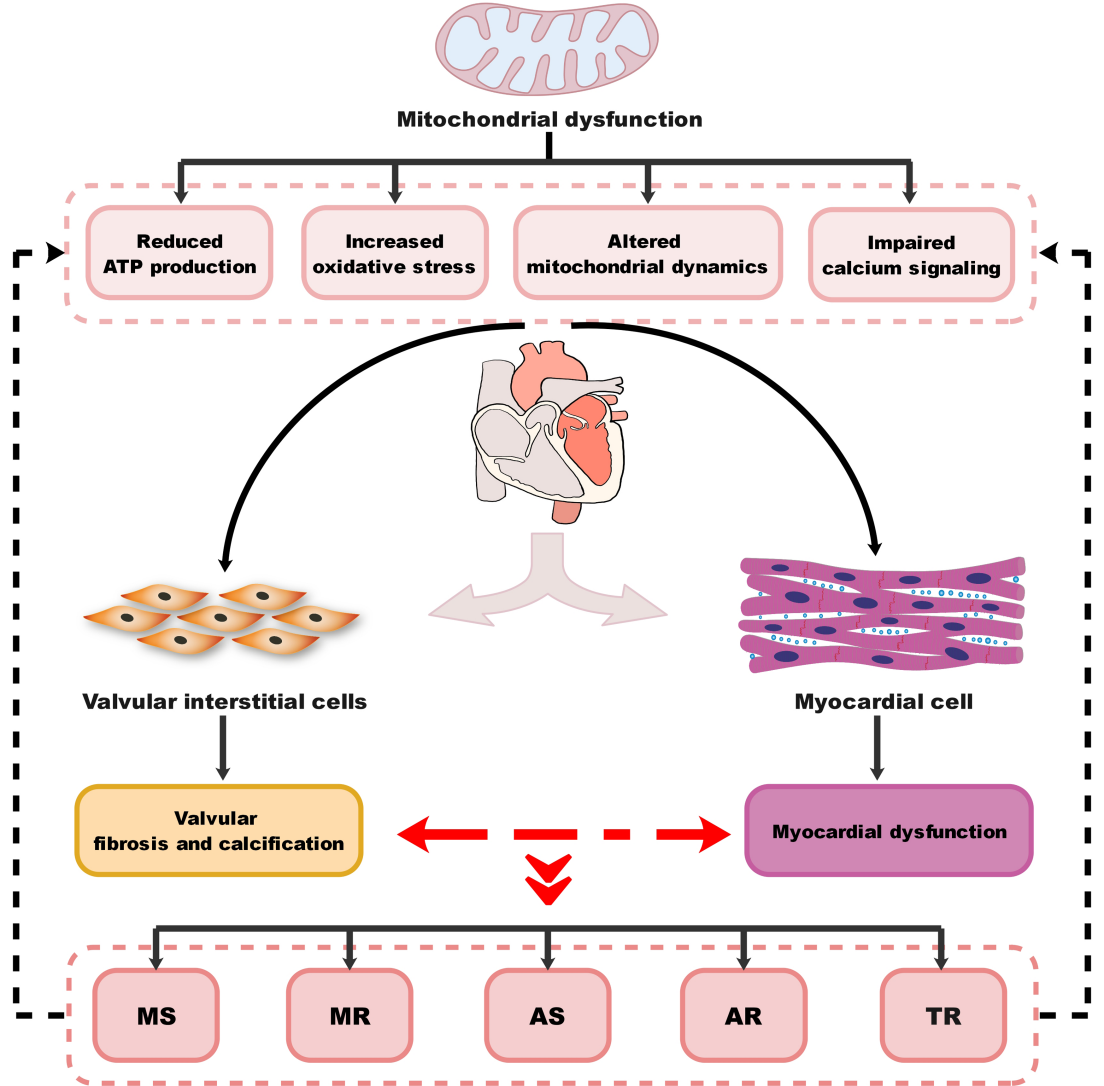
Diagram of the association between MitD and VHD. MitD, mitochondrial dysfunction; VHD, valvular heart disease; MS, mitral stenosis; MR, mitral regurgitation; AS, aortic stenosis; AR, aortic regurgitation; TR, tricuspid regurgitation.

VHD can lead to HF through multiple mechanisms, including both mechanical damage to the valve itself and the impact of valve lesions on the myocardium. The interactions between different valve damages and myocardium dysfunction can exacerbate these processes and lead to the development of different VHD-related HF subtypes. MitD may play an essential role in this process and also contribute to the progression and severity of the condition. MitD can lead to reduced ATP production, increased oxidative stress, impaired calcium signaling, and altered mitochondrial dynamics. Different valvular damages lead to altered blood flow (Figure 1), causing the heart to work harder in pumping blood, resulting in increased oxygen and energy demand. This increased demand on the heart can further exacerbate MitD in valve interstitial cells (8, 101) and myocardial cells (102). On the other hand, MitD can contribute to the development and progression of VHD by promoting valvular fibrosis and/or calcification as well as myocardial dysfunction. This forms a vicious cycle (Figure 2) that promotes the deterioration of impaired cardiac function.

Cardiac mitochondria are essential for contractile function, while a progressive impairment of mitochondrial morphology and function characterizes HF (103). Different valvular damages led to the changes of mitochondrial morphology and function. Thiedemann and Ferrans (104) previously showed that the fibrotic areas of LA exhibited degenerative changes of varying severity (including variations in size and number of mitochondria, occurrence of abnormal mitochondria) in 14 patients with mitral valvular disease. Compared with those with pure MS, the severity of degeneration was much worse in MR patients with or without MS. Maron et al. (105) also showed that the degenerated myocardium (e.g., proliferation of mitochondria) was present in the marked fibrosis areas of LV in 16 patients with aortic valvular disease. Calcific AS phenotype displayed the characteristics of defects in mitochondrial quality control mechanisms (106). Similarly, compared with those with pure AS, the severity of degeneration was greater in AR patients with or without AS, even in the early stage of AR (107). In a mouse model of fibrocalcific AS, Roos et al. (108) found that maintenance of mitochondrial antioxidant capacity was important to prevent the progression of AS. A recent study on the identification of the mitochondrial DNA haplogroups in patients with AS by Serrano-Teruel et al. (109) found that mitochondrial DNA haplogroups may be correlated with the severity of AS.

Mitochondrial function was disordered with its morphological changes. The changes of mitochondrial morphology and function represented early cardiac adaptation to pressure overload or volume overload of LA and/or LV. In patients with ostium secundum atrial septal defect, Macchiarelli et al. (110) reported that this was not only a subcellular sign of myocardial hypertrophy (e.g., increased number of mitochondria) but also focal degenerative changes (e.g., rupture of mitochondrial cristae) because of RA volume overload. In patients with ventricular septal defect or endocardial cushion defect, Pham et al. (111) also found that myocardial hypertrophy may be secondary to RA volume overload, whereas degenerative changes may be secondary to RA pressure overload. In a mouse model of short-term hypertension, Aguas et al. (112) showed that short-term and moderate LA and LV pressure overload induced adaptive changes (e.g., moderate mitochondrial enlargement) in left atrial cells at a stage when ventricular cells have morphological characteristics close to normal cells. Ulasova et al. (113) constructed a mouse model of LV volume overload induced by aortocaval fistula to mimic the AR-related LV volume overload status and found that the mitochondrial state 3 respiration of subsarcolemmal mitochondria decreased by 40%. Schwarzer et al. (114) constructed a mouse model of LV pressure overload induced by transverse aortic constriction to mimic the AS-related LV pressure overload status and found that the pressure overload significantly impaired respiratory rates of interfibrillar mitochondria, related to the reduction of total mitochondrial content and total mitochondrial volume density. In a dog model of AS, Wollenberger and Schulze (115) found that mitochondrial morphology changed between failing and normal myocardial tissues. The mitochondrial activity (e.g., mitochondrial respiratory chain enzyme complexes I + III) increased with increasing aortic valve pressure gradient in AS patients (116). Volume overload increased the vulnerability of cardiac mitochondria without affecting their functions in the absence of pressure overload, at a time when harmful cardiac remodeling was observed but systolic dysfunction and decompensation had not yet occurred (117).

Different valvular damages caused by mitochondrial dysfunction were associated with the development of HF. Chang et al. (118) found that atrial mitochondrial dysfunction was associated with MR- and TR-induced HF. Ahmed et al. (119) reported that the preserved ventricular function in degenerative MR patients receiving early surgery was associated with mitochondrial damage-induced oxidative stress. The mitochondrial unfolded protein response (UPR^mt^) played a key role of maintaining mitochondrial function. Smyrnias et al. (103) found that the UPR^mt^ of the myocardial tissue was activated in the mice model of AS and in patients with AS, which may reverse AS-induced HF (120, 121).

In addition to the homogeneity of MitD between HFpEF and HFrEF, there was heterogeneity on MitD between the two subtypes of HF. Hunter et al. (122) found that impairment in peripheral mitochondria in patients with HFpEF was greater than that in patients with HFrEF. In particular, the phenomenon was also observed in VHD-related HF. Moorjani et al. (123) showed that cardiomyocyte mitochondrial dysfunction was associated with the development of HFpEF, HFmrEF, or HFrEF due to AR-related LV volume overload. In the myocardium from the patients with HFpEF or HFrEF due to valvular damages, compared with those with normal left ventricular function, mitochondrial fragmentation and cristae destruction were evident, and mitochondrial area was decreased in HFpEF. These mitochondrial morphological changes were more pronounced in HFrEF (124).

## 6. Limitation and prospects

First, the mechanism of VHD-related HF involves not only mechanical damages to the valve itself but also valve damages caused by myocardial ischemia, which interact to drive the development of VHD-related HF. Based on the heterogeneity of the types of valve damage, the mechanisms of the different valve damages on different phenotypes of VHD-related HF are still unclear, especially the MS- or AR-related HFpEF phenotypes. The prevalence of tricuspid valve damage is relatively high, but single tricuspid valve surgery is still rare, which increases the difficulty of obtaining tricuspid valve samples. Therefore, specific animal models are urgently needed to investigate the relationship between tricuspid valve damage and HF. The echocardiographic assessment and clinical implication of functional TR in HF with reduced or preserved EF (ECLIPSE-HF) study (NCT05209919) is a non-interventional, prospective, international, multicenter, longitudinal study designed to characterize the pathophysiological mechanisms and clinical relevance of functional TR in HFrEF, HFmrEF, and HFpEF (125).

Second, the current research evidence focuses on single-valve/single-lesion induced HF, ignoring the fact that clinically VHD patients often suffered from combined valve damages. A study based on etiology, clinical features, treatment, and outcomes of VHD in a Chinese population has been preliminarily completed (NCT03484806). Furthermore, the development of VHD-related HF and its prognosis depend on the spatial (combination of different valvular lesions) and temporal effects (sequence of valvular lesions) of valvular damages, which complicates the exploration of VHD. In particular, HFpEF is a heterogeneous syndrome. Previous studies on HFpEF mostly focused on the effect of different etiologies caused by primary and/or secondary myocardial ischemia in HFpEF development and ignored the role of valve damages and PH degree in it, making the heterogeneity of the VHD-related HFpEF phenotype more pronounced. It would be beneficial to characterize the VHD phenotype based on a three-dimensional model of valvular damage-myocardial ischemia-pulmonary pressure. Hence, a real-world study on VHD-related HF phenotypes based on artificial intelligence is one of the promising new directions.

Third, the right heart acts as a separate system, independent of the left heart. RVD is almost always associated with poor prognosis of HF. However, the role of RVD which has an independent HF effect in various HF subtypes has been overlooked. RVD is the result of the combined effect of three factors, such as tricuspid valve damage, RV myocardial ischemia, and PH degree.

Lastly, whether MitD is a cause or a consequence of VHD remains unclear. Evidence indicates that MitD may play an important role in various valvular damages and its related HF subtypes, but understanding of the specific molecular mechanisms of MitD in valvular damages derived from HF subtypes still faces many challenges, particularly in the processes of inflammation and oxidative stress (8, 108). By targeting cardiac fibrosis, the endothelial-to-mesenchymal transition is critical to valve development and valve tissue homeostasis (126) as well as HF (127) and involves the activation and phenotypic conversion of valvular endothelial cells and valvular interstitial cells (128). The effect of MitD in endothelial-to-mesenchymal transition needs further understanding. On the other hand, VHD can also lead to MitD by altering cellular metabolism and reducing the availability of oxygen and nutrients. Overall, the exact relationship between MitD and VHD needs further elucidation.

## 7. Conclusion

VHD often coexists with HF in clinical practice, especially in the elderly. Due to the spatial (combination of different valvular lesions) and temporal effects (sequence of valvular lesions) of valvular HF, this can complicate the patient's condition. In addition, physicians may deal with a dilemma when deciding on a treatment plan. Some classic prognosis-improving medications in patients with non-valvular HF are not applicable in patients with VHD due to some contraindications, or they may fail to improve clinical outcomes in patients with valvular HF. This suggests a partial lack of understanding of the mechanism of occurrence and development of VHD-related HF. Further studies are necessary to fully comprehend the complex interactions in the pathophysiology and pathogenic mechanisms of different HF subtypes with various valvular damages. This will assist in developing optimal diagnostic and therapeutic strategies for this special population.

Since MitD acts an essential role in the pathogenesis of VHD and HF, further investigations are encouraged to reveal the relationship of MitD with the morbidity and mortality in VHD-related HF (e.g., HFrEF, HFmrEF, and HFpEF) through large-scale, real-world clinical studies. These studies should aim to clarify the homogeneity and heterogeneity of MitD among the various phenotypic subgroups of VHD. Based on these studies that have been planned and conducted, mitochondrial targeting may lead to the development of early and accurate diagnosis and/or treatment strategies, which could result in preferable outcomes for patients with VHD-related HF.
